# Flavonoids of *S. suberectus Dunn* Regulate Cyclophosphamide-Induced Immunosuppression Through NF-κB Pathways

**DOI:** 10.3390/vetsci12080762

**Published:** 2025-08-16

**Authors:** Jinwu Zhang, Bo Zhang, Shiqi Huang, Jianhao Deng, Yiying Liang, Jiakang He, Tingjun Hu, Liji Xie, Hailan Chen, Meiling Yu

**Affiliations:** 1 Guangxi Key Laboratory of Animal Breeding, Disease Control and Prevention, College of Animal Science and Technology, Guangxi University, Nanning 530004, China; 2018302041@st.gxu.edu.cn (J.Z.);; 2 Guangxi Key Laboratory of Veterinary Biotechnology, Guangxi Veterinary Research Institute, Nanning 530001, China

**Keywords:** metabolomics, immunosuppression, total flavonoid of *Spatholobus suberctus Dunn*, network pharmacology, NF-κB

## Abstract

This study investigated the immunomodulatory effects and underlying mechanisms of total flavonoid of *Spatholobus suberectus Dunn* (TFSD) and its primary constituent formononetin (FMN) in immunosuppressed mouse models. *Spatholobus suberectus Dunn* (*S. suberectus Dunn*) was first analyzed qualitatively and quantitatively by broadly targeted metabolomics using Ultra-performance liquid chromatography-tandem mass spectrometry (UPLC-MS/MS). Integrated network pharmacology and molecular docking approaches were used to investigate the potential mechanisms underlying *S. suberectus Dunn*’s immunomodulatory effects. Subsequently, mice were pretreated with TFSD and FMN for seven days before constructing an immunosuppression model through intraperitoneal injection of 200 mg/kg.bw cyclophosphamide (CTX). In vivo experiments validated the findings and investigated the mechanisms underlying the effects of TFSD and FMN on immunosuppression. Metabolomic analysis identified 501 distinct flavonoids in *S. suberectus Dunn*, with FMN exhibiting the highest relative abundance among all detected compounds. The primary active components of *S. suberectus Dunn* against immunosuppression are flavonoids, including FMN and vestitol. The core targets of these components were identified as NF-κB and IKKβ, with the NF-κB signaling pathway being suggested as the most probable mechanism of action. FMN exhibited strong binding affinity to the core targets, NF-κB p65 and IKKβ. In vivo experiments indicated that pretreatment with TFSD and FMN prevented CTX-induced pathological damage in the spleen and thymus and increased immune cell counts and immunoglobulin levels. Additionally, it significantly upregulated the secretion and expression of key cytokines and the mRNA expression of NF-κB p65, IKKα, and IKKβ (*p* < 0.05 or *p* < 0.01). In conclusion, TFSD and FMN can protect mice from CTX-induced immunosuppression by regulating the NF-κB signaling pathway, making them promising drug candidates for preventing and treating immunosuppression-related diseases.

## Simple Summary

Immunosuppressive diseases are frequently encountered in animal husbandry, including porcine reproductive and respiratory syndrome, swine fever, and infectious bursal disease in chickens. Due to their high mortality rates, these diseases pose a significant threat to the healthy development of animal husbandry. The total flavonoids of *Spatholobus suberectus Dunn* (TFSD) exhibit significant immunomodulatory properties, demonstrating their therapeutic potential against immunosuppressive conditions. The study results indicate that TFSD administration significantly mitigates cyclophosphamide-induced immunotoxicity by preserving splenic and thymic architecture while restoring immune competence in treated subjects. Additionally, network pharmacology, molecular docking, and in vivo experiments demonstrated that TFSD can reduce immunosuppression-induced damage by regulating the NF-κB signaling pathway. These findings suggest that TFSD is a promising immunotherapeutic candidate for managing immunosuppression-related immune dysfunction in veterinary medicine.

## 1. Introduction

The immune system, which includes the humoral and cellular immunity, is an important defense mechanism for animals against pathogenic microorganisms (including viruses) and malignant cells. It is of great significance for maintaining health [1,2]. However, many livestock diseases can cause immune system disorders and immunosuppression in animals, including swine fever, porcine reproductive and respiratory syndrome, and poultry-related diseases [3,4]. Classical swine fever is caused by classical swine fever virus (CSFV), which damages the pig’s immune system, inhibits the lymphocyte’s (LYM) proliferation and differentiation, reduces the immune function, and causes severe immune suppression symptoms in infected pigs [5]. The porcine reproductive and respiratory syndrome virus (PRRSV), an arterivirus, primarily targets and depletes pulmonary alveolar macrophages in swine. This specific immunopathology results in significant immunosuppression, compromising both innate and adaptive immune responses in affected animals. Additionally, infection causes severe immune suppression in affected pigs [6]. When immunocompromised pigs are infected with CSFV or PRRSV, pregnant sows can experience miscarriages, stillbirths, and mummified fetuses. The mortality rate among newborn piglets can exceed 90%, and those that survive can become lifelong carriers of the virus, posing significant safety risks to pig farms [7]. Infectious bursal disease virus (IBDV), a member of the Birnaviridae family, is the etiological agent of infectious bursal disease, which is an acute, highly contagious immunosuppressive disorder in poultry. IBDV replicates significantly in lymphoid organs, initially triggering an acute inflammatory response, which subsequently results in atrophy of the bursa of Fabricius and severely impairs immune function [8]. Chicken infectious anemia virus (CIAV), an avian anellovirus, induces immunosuppressive anemia through targeted lymphocytolysis in poultry. CIAV primarily affects the hematopoietic tissue of the bone marrow and the immune system, which results in a decrease in the number of LYM in the lymphoid organs, the inhibition of antibody production, and cellular immune dysfunction [9]. Immunosuppression reduces the immune function of chickens, resulting in reduced resistance to various pathogenic microorganisms and the development of secondary infections. In addition, it results in slow growth, slow weight gain, high feed conversion rates and mortality rates, reduced production efficiency, decreased egg production and egg quality, and thinner eggshells, thereby significantly impacting poultry farming [10]. Therefore, immunosuppression reduces the body’s resistance to endogenous and exogenous immunogens, resulting in disease progression, secondary infections, and vaccine failure, causing significant losses to the livestock industry [11,12].

Immunosuppression is not limited to farm animals; chemotherapy can also induce immunosuppression in companion animals, including dogs and cats. The incidence of tumors in pets is on the rise each year. Cyclophosphamide (CTX) has been included in the “List of Human Medicines Urgently Needed for Clinical Use in Pets (61 types)” by the Ministry of Agriculture and Rural Affairs of China. CTX is an important anticancer drug, particularly in the treatment and management of tumors, including multiple myeloma and breast cancer. It exerts its antitumor effect by forming cross-links within DNA, thereby inducing cell apoptosis [13]. As a non-specific cell cycle inhibitor, it can indiscriminately suppress proliferating immune cells, potentially resulting in paradoxical inflammatory responses and subsequent immunosuppression [14].

Since CTX can cause immunosuppression, researchers frequently use it to induce immunosuppression models to investigate the mechanisms of immunosuppression and drug development [15]. Li et al. [16] used CTX to establish a mouse immunosuppression model to investigate the regulatory effects of ginsenosides on immunity, microbiota, and metabolism. They demonstrated that CTX can increase host immunity through intestinal microbiota modulation, metabolic homeostasis restoration, and TLR4/MyD88/NF-κB pathway activation. Consequently, a theoretical foundation for clinical translation was established. The CTX-induced murine immunosuppression model serves as a valuable preclinical platform for evaluating immunomodulatory drug candidates, enabling both safety assessment and efficacy optimization prior to clinical translation [17].

*Spatholobus suberectus Dunn* (*S. suberectus Dunn*) is an herb rich in flavonoids. Its extracts contain various bioactive compounds, including flavonoids, lignans, and anthraquinones. Among these, flavonoids are the key components in the alcoholic extract of *S. suberectus Dunn* [18]. In our previous study, we demonstrated that the total flavonoid content (TFSD) extracted from *S. suberectus Dunn* using ethanol was as high as 45.29% [19]. In mice infected with *Porcine circovirus* type 2 and Escherichia coli, chicken blood vine extract exhibited a significant promotion of bone marrow hematopoietic function and strong immunomodulatory effects [20]. Although there is no commercial immunomodulator for this plant species, multiple studies have reported that *S. suberectus Dunn*’s extract has immunomodulatory effects. Chen et al. [21] found that adding 1.00% extract of *S. suberectus Dunn* to the diet can significantly improve the growth performance and immune function of Lingshan Ma chickens. Yang et al. [22] found that *S. suberectus Dunn* extract from ethyl acetate can effectively promote NK cell proliferation and has immunomodulatory effects. Additionally, *S. suberectus Dunn* has a complex chemical composition and multiple pharmacological activities. However, its clinical application is restricted by the inability to fully elucidate its specific mechanism of action [23]. Using metabolomics and network pharmacology is beneficial for analyzing the primary active ingredients of Chinese herbal medicines and investigating their mechanisms of action. Li et al. [24] demonstrated that tricin, the bioactive flavonoid in Wei Jing Tang, suppresses non-small cell lung cancer progression through dual inhibition of PRKCA/SPHK/S1P and anti-apoptotic signaling pathways. This study used the aforementioned techniques to analyze the composition of *S. suberectus Dunn*, systematically investigating the immunoregulatory effects of TFSD and its primary component formononetin (FMN), as well as their mechanisms of action. Initially, comprehensive, broadly targeted metabolomics profiling was performed to characterize the principal bioactive constituents of *S. suberectus Dunn*. Integrated network pharmacology and molecular docking strategies were used to predict *S. suberectus Dunn*’s putative immunomodulatory mechanisms. Finally, in vivo validation studies determined the immunomodulatory mechanisms of TFSD and FMN in the CTX-induced immunosuppression model, establishing a pharmacological foundation for developing novel immunotherapeutics.

## 2. Materials and Methods

### 2.1. Reagents

The dried herbal slices of *S. suberectus Dunn* were obtained from the Gaoxiong Chinese Medicine (Nanning, China), with their origin traced to Chongzuo City, Guangxi. The plant material was authenticated as genuine *S. suberectus Dunn* by the Pharmacological Laboratory of the College of Animal Science and Technology at Guangxi University [25]. The laboratory of Pharmacology and Toxicology of Guangxi University provided 10 mg/mL of TFSD, which was extracted from *S. suberectus Dunn* using the ethanol extraction method as described by Fu et al. [19]. Moreover, formononetin (FMN, B2426324), cyclophosphamide (CTX, K2217399), and quercetin (QCT, C14950778) were obtained from Shanghai Aladdin Biochemical Technology Co., Ltd. (Shanghai, China). ELISA kits of interleukin-2 (IL-2, 109237), tumor necrosis factor-ɑ (TNF-α, 104395), immunoglobulin G (IgG, 105830), and IgM (106597) were obtained from Shanghai Yiyan Biological Technology Co., Ltd. (Shanghai, China). All-In-One 5 × RT MasterMix (G592) and BlasTaq™ 2 × qPCR MasterMix (G891) were obtained from Applied Biological Materials Co., Ltd. (Richmond, BC, Canada). PCR amplification primers were custom-synthesized by Sangon Biotech Co., Ltd. (Shanghai, China).

### 2.2. S. suberectus Dunn Sample Pretreatment

*S. suberectus Dunn* samples were initially vacuum freeze-dried using the Scientz-100F lyophilizer. The dehydrated material was then pulverized into fine powder using a Retsch MM 400 grinder (30 Hz, 90 s). Powdered samples (50 mg) were extracted with 1.2 mL of ice-cold 70% methanol (containing internal standards) at a 24:1 solvent-to-sample ratio (*v*/*w*). The mixture was mixed by intermittent vortexing (30 s pulses at 30 min intervals, repeated six times) to ensure complete extraction. After centrifugation (12,000× *g*, 3 min), supernatants were membrane-filtered (0.22 μm) and aliquoted into UPLC-MS/MS vials for analysis.

### 2.3. UPLC Conditions

Samples were analyzed on an ExionLC™ AD UPLC-ESI-MS/MS system equipped with an Agilent SB-C18 column (2.1 × 0.1 cm, 1.8 µm; 40 °C). The mobile phase comprised (A) 0.1% aqueous formic acid and (B) 0.1% formic acid in acetonitrile. The gradient program comprised 95% A/5% B initial conditions, a linear gradient to 5% A/95% B over 9 min, a 1 min hold, return to 95% A in 1.1 min, and 2.9 min re-equilibration. The mobile phase flow rate was set to 0.35 mL/min with 2 μL injections. Column effluent was analyzed by ESI-QTRAP-MS for tandem mass spectrometry detection.

### 2.4. ESI-QTRAP-MS/MS

Mass spectrometry was performed in electrospray ionization (ESI) mode with optimized parameters: source temperature 500 °C; ion spray voltage ±5500 V (positive/negative polarity); and gas pressures of 50 psi (nebulizing), 60 psi (turbo), and 25 psi (curtain). Quantitative analysis was performed in multiple reaction monitoring (MRM) mode using nitrogen collision gas at medium pressure, with collision-activated dissociation maintained at high intensity. For each target metabolite, specific MRM transitions were monitored during their corresponding elution windows, with individually optimized declustering potentials and collision energies to ensure maximum sensitivity.

### 2.5. Acquisition of Target Data

The Traditional Chinese Medicine Systems Pharmacology Database and Analysis Platform (TCMSP, http://tcmspw.com/tcmsp.php, accessed on 12 September 2024) represents a specialized bioinformatics resource for systematic investigation of herbal medicine components, their targets, and associated pharmacological networks. [26]. The screening conditions for all components of *S. suberectus Dunn* from TCMSP were oral bioavailability (OB) ≥ 30% and drug-likeness (DL) ≥ 0.18. Corresponding components and target proteins of *S. suberectus Dunn* were then obtained. Target genes were identified and standardized using the UniProt database (https://www.uniprot.org/, accessed on 13 September 2024) [27]. Network analysis of bioactive compounds and their molecular targets was performed using Cytoscape 3.9.1 to investigate potential pharmacological interactions.

Immunosuppression-related disease targets were identified through systematic searches of the PharmGKB (https://www.PharmGKB.org/, accessed on 14 September 2024) and GeneCards (https://www.genecards.org/, accessed on 14 September 2024), followed by intersectional analysis with *S. suberectus Dunn* compound targets.

### 2.6. Network Construction

Potential immunosuppressive targets of *S. suberectus Dunn* were identified by analyzing target intersections through Venny plot visualization (Microbiotics platform). Gene candidates identified through intersection analysis were subjected to protein–protein interaction (PPI) network construction using STRING (confidence score ≥ 0.9; unconnected nodes removed, https://cn.string-db.org/, accessed on 15 September 2024) [28]. The network was analyzed in Cytoscape 3.9.1 (TSV format) using topological metrics (Degree, Closeness, and Betweenness), with key targets defined as nodes exceeding median centrality values [29]. This systematic approach identified hub targets within the PPI network.

Intersecting genes were analyzed using gene ontology (GO) and Kyoto encyclopedia of genes and genomes (KEGG) pathway enrichment analyses through DAVID (https://david.ncifcrf.gov/tools.jsp, accessed on 15 September 2024) [30]. The analysis identified biological processes (BP), cellular components (CC), molecular functions (MF), and significantly enriched pathways [31]. The results were visualized using bioinformatics tools.

### 2.7. Molecular Docking Studies

Based on the network pharmacology analysis, FMN was identified as one of the key bioactive components of *S. suberectus Dunn* for treating immunosuppression, with the highest concentration in the TFSD. PPI network and enrichment analyses predicted that *S. suberectus Dunn* can exert its immunomodulatory effects through the NF-κB signaling pathway. Consequently, FMN was selected for molecular docking with two key target proteins in the PPI network: NF-κB p65 (the highest-ranked target) and IKKβ (the third-ranked target). The FMN ligand was used in the molecular docking protocol against NF-κB p65 and IKKβ receptor structures (PDB, http://www.rcsb.org/, accessed on 18 September 2024). The three-dimensional structure of FMN was retrieved in SDF format (TCMSP, PubChem), converted to MOL2 (Open Babel 2.4.1), and energy-minimized for docking studies. Docking simulations were performed with AutoDock Vina [32] using the PyMol 5.2.7 interface, with binding affinities evaluated based on minimal binding energy to predict ligand-receptor interaction efficiency.

### 2.8. Animals and Treatments

All animal procedures were approved by the Institutional Animal Care and Use Committee of Guangxi University (Approval No. GXU-2022-210, date: 10 March 2022) and conducted following NIH animal care guidelines (1996). Authenticated *S. suberectus Dunn* specimens (origin: Chongzuo City, Guangxi) were obtained from the Gaoxiong Chinese Medicine (Nanning, China) and verified by Guangxi University’s Pharmacological Laboratory [25]. The samples were ground to medium-fine powder and extracted with 50% ethanol (1:30 *w*/*v*, 80 °C, 3 h). The filtrate was rotary-evaporated (60 °C, 50 rpm) and lyophilized to yield TFSD as a tan powder. FMN (B2426324) was purchased from Shanghai Aladdin Biochemical Technology Co., Ltd. (Shanghai, China). TFSD and FMN were freshly prepared as suspensions at required concentrations by dissolving in PBS immediately before administration. Based on our previous studies using TFSD (25–100 mg/kg.bw) in CTX-induced liver injury models [33] and FMN (25–100 mg/kg.bw) in *E. coli*-induced sepsis models [20]. Both compounds exhibited significant therapeutic effects at these dose ranges. FMN is the predominant (though not exclusive) bioactive component in TFSD and has been demonstrated to increase immunity in H22 hepatoma-bearing mice at doses of 10–50 mg/kg.bw [34]. Therefore, we selected FMN doses of 12.5, 25, and 50 mg/kg.bw for this study.

Furthermore, 90 SPF Kunming mice (4-week-old, 14–18 g; equal sex distribution) were obtained from Guangxi Medical University’s Laboratory Animal Center. Following a one-week acclimation under standard SPF conditions, mice were randomly divided into nine experimental groups: Control, CTX, QCT, and three dose-level groups each for TFSD (High/Medium/Low) and FMN (High/Medium/Low). Mice were orally administered with normal PBS, QCT, and different doses of TFSD or FMN for seven consecutive days according to Table 1. After 4 h of the last drug administration, mice were intraperitoneally injected with CTX in a dose of 200 mg/kg.bw to establish immunosuppressed models. The clinical status of mice was observed and recorded for eight days after the first administration.

### 2.9. Measurement of Body Weights and Immune Organs Indexes

A stainless-steel feeding needle (diameter 1.2 mm, length 3 cm) was used to administer drugs orally through gavage. After 4 h fast, the mice were gently restrained, and the needle was inserted into the stomach through the esophagus at a 30° angle. The suspension was administered at 10 mL/kg.bw for 10 s, followed by 3 s dwell time. At 24 h post-gavage, mice were weighed and humanely euthanized by sodium pentobarbital overdose (200 mg/kg, i.p.). The immune organs (spleen and thymus) were immediately harvested and weighed for subsequent analysis.

### 2.10. Histopathological Observation

Representative spleen and thymus samples (2–3 mm thick) were formalin-fixed, paraffin-embedded, and sectioned at 5 μm (rotary microtome) for Hematoxylin and Eosin (H&E) staining and histopathological analysis (Olympus BX-FM microscope). Histopathological evaluation was performed using a microscope (BX-FM; Olympus Corp., Tokyo, Japan).

### 2.11. Hematology Analysis

Terminal blood was collected through retro-orbital venous plexus puncture under anesthesia by inserting a heparinized capillary tube (1 mm diameter) through the medial canthus into the orbital sinus and stored at 4 °C before analysis. For complete blood count measurements, 100 μL aliquots were processed using an automated hematology analyzer according to the manufacturer’s protocol. Complete blood counts were performed using a DxH800 hematology analyzer (Beckman Coulter, Inc., Brea, CA, USA), quantifying white blood cells (WBC), red blood cells (RBC), hemoglobin (HGB), platelets (PLT), LYM, and granulocytes (GRAN).

### 2.12. ELISA Assay

For serological analysis, whole blood samples (200 μL) were collected according to the manufacturer’s protocol for the ELISA assay. Serum was separated from blood by centrifugation (4000× *g*, 10 min, room temperature) and stored at −80 °C. IgG, IgM, IL-2, and TNF-α levels were quantified using commercial ELISA kits (Yiyan Biological Technology Co., Ltd., Shanghai, China) following the manufacturer’s guideline. The absorbance was read on a PerkinElmer Multimode Plate Reader (PerkinElmer Instruments, Schwerzenbach, Switzerland).

### 2.13. RT-qPCR Assay

Splenic mRNA expression levels of IL-2 and TNF-α were measured through RT-qPCR. Total RNA was extracted (RNAprep Pure Cell Kit, Tiangen, Beijing, China) and reverse transcribed into cDNA (1 μg RNA input; All-In-One 5 × RT MasterMix). Quantitative PCR was performed in 20 μL reactions using BlastTaq™ 2 × qPCR MasterMix using an ABI 7500 Fast system. The cycling parameter conditions were as follows: 95 °C for 15 min; 40 cycles of 95 °C for 10 s, 60 °C for 30 s, and 72 °C for 32 s (fluorescence acquisition). Gene expression was quantified using the 2^−ΔΔCt^ method (primer sequences are presented in Table 2).

### 2.14. Statistical Analysis

Data are presented as mean ± standard error of mean (SEM). Statistical significance was determined by one-way analysis of variance with a least significant difference post hoc test. The data were analyzed using the Statistical Package for Social Sciences software (version 21.0). *p* < 0.05 and 0.01 were considered significant and highly significant, respectively.

## 3. Results

### 3.1. Metabolomics Results of S. suberectus Dun

**Figure 1:**
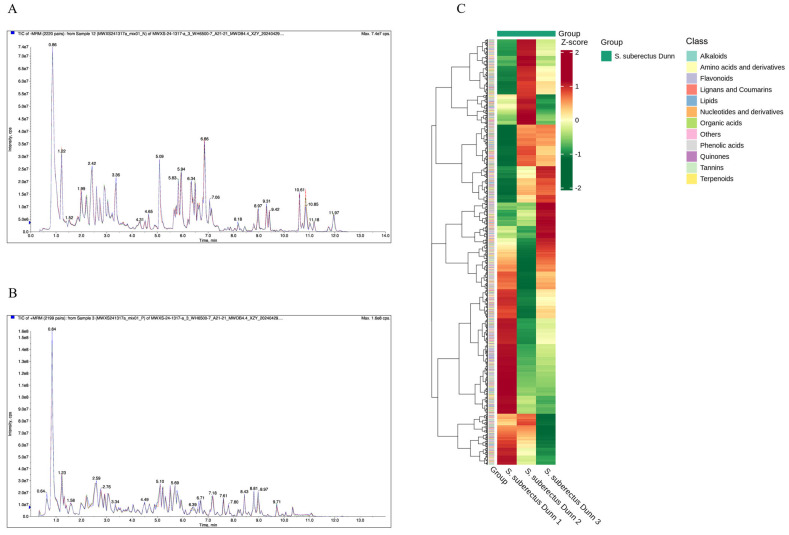
Metabolomics analysis of *S. suberectus Dunn*. (**A**) TIC chromatogram overlaps of QC samples (negative ion mode). (**B**) TIC chromatogram overlaps of QC samples (positive ion mode). (**C**) Cluster heat maps of all metabolites.

Broad-targeted metabolomic profiling through UPLC-MS/MS enabled comprehensive quantification of *S. suberectus Dunn* metabolites. Repeated quality control (QC) injections exhibited highly reproducible total ion current (TIC) chromatograms (Figure 1A,B), confirming excellent instrumental stability. The consistent retention times and peak intensities confirmed robust system performance throughout the analytical sequence. A total of 2077 metabolites of *S. suberectus Dunn* were identified, including 501 flavonoids, 319 others, 238 amino acids and their derivatives, 190 lipids, 189 phenolic acids, 139 alkaloids, 123 terpenoids, 111 organic acids, 100 lignans and coumarins, 81 nucleotides and their derivatives, 64 quinones, and 21 tannins (Figure 1C). Among the top ten relative contents of *S. suberectus Dunn* metabolites, there were seven flavonoids, and the relative content of FMN was the maximum (Table 3).

### 3.2. Compound-Target Network and PPI Network Construction

**Figure 2:**
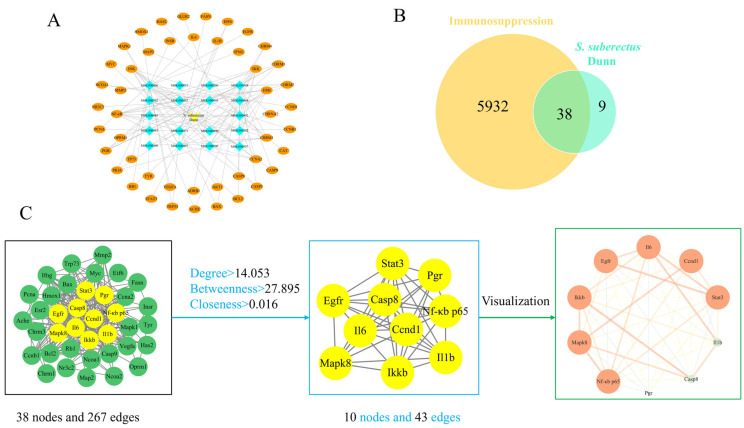
Network pharmacological analysis of *S. suberectus Dunn* and immunosuppression. (**A**) Interaction network of *S. suberectus Dunn* components-targets. Yellow inverted triangles represent *S. suberectus Dunn*, blue diamonds represent active components (n = 18), and orange ovals represent target genes (n = 47). (**B**) Target overlap between *S. suberectus Dunn* and immunosuppression-associated genes. (**C**) The PPI network of shared targets. Nodes represent proteins. Green and yellow nodes are 38 intersecting proteins. The yellow nodes (n = 10) are filtered by setting a median greater than Degree, Betweenness, and Closeness. Edge colors indicate protein-binding affinity (blue: weak; orange: strong).

Combined with the results of TCMSP, *S. suberectus Dunn* was screened and identified 24 active components and 47 targets, listing the top 10 active components in terms of OB value (Table 4). The *S. suberectus Dunn* components-targets network, obtained by importing drug components and targets into Cytoscape 3.9.1, comprised 66 nodes and 136 edges (Figure 2A).

GeneCards and PharmGKB databases yielded 5247 and 950 immunosuppression-associated gene targets, respectively. After removing the duplicates, 5970 immunosuppression target genes were obtained. After mapping the targets of *S. suberectus Dunn* and immunosuppression with Venny 2.1, 38 intersecting targets were obtained (Figure 2B). The PPI network, constructed in Cytoscape 3.9.1 and analyzed with CentiScaPe 2.2, included 38 nodes and 267 edges pre-filtering. After applying Degree, Betweenness, and Closeness thresholds, this was refined to 10 nodes and 43 edges. The key targets of *S. suberectus Dunn* acting on immunosuppressive diseases are primarily NF-κB p65 and IKKβ (Figure 2C).

### 3.3. GO Enrichment, KEGG Pathway Analysis, and Molecular Docking Results

**Figure 3:**
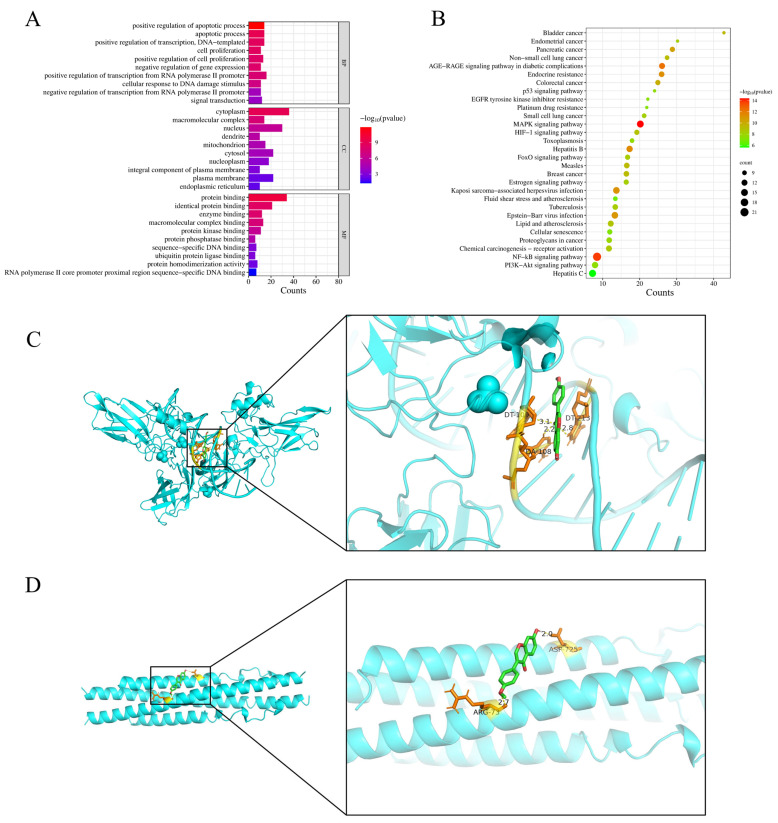
Network pharmacology prediction for *S. suberectus Dunn* treatment of immunosuppression. (**A**) GO enrichment analysis for targets of *S. suberectus Dunn* against immunosuppression (top 10). (**B**) The bubble chart of KEGG pathway analysis (Top 30). Node size corresponds to gene count; color intensity indicates pathway significance (−log10[*p*-value]). Molecular docking of FMN with core proteins. (**C**) FMN-NF-κB p65, (**D**) FMN-IKKβ.

GO enrichment analysis identified the top 10 significantly enriched terms in each category: BP included transcription regulation through RNA polymerase II and cellular proliferation; CC comprised nuclear and membrane-associated structures; while MF involved transcriptional regulation and gene expression modulation (Figure 3A). KEGG analysis identified the top 30 enriched pathways, including NF-κB, MAPK, and PI3K-Akt signaling pathways (Figure 3B). Additionally, the *p*-value and count value of the NF-κB signaling pathway were more significant compared to the other pathways, followed by the MAPK signaling pathway. These results suggest that the mechanism of *S. suberectus Dunn* against immunosuppressive diseases can involve the NF-κB signaling pathway.

Docking scores quantitatively reflect ligand-target binding affinity and complex stability, with absolute values ≥ 4.25, >5.0, and >7.0 representing threshold, good, and strong binding activity, respectively [35]. The binding energies of NF-κB p65 and IKKβ to FMN were −6.06 and −5.04 kcal/mol, respectively. The absolute values of their binding energies were >5.0, indicating good binding activity between the ligand and the receptor. The depictions of the structural binding interfaces and corresponding calculated binding energies for each protein–ligand complex, with energetically favorable interactions prominently identified, are represented in Figure 3C,D.

### 3.4. TFSD and FMN Ameliorated the Clinical Performance of CTX-Administered Mice

During the seven days of TFSD and FMN pretreatment, all mice were in a good mental state, exhibiting active behavior, bright hair, and a normal appetite for drinking and eating. At 12 h post intraperitoneal CTX injection, mice exhibited characteristic clinical manifestations of immunosuppression, including lethargy, dispiritedness, and anorexia. However, TFSD and FMN pretreatment alleviated these symptoms in immunosuppressed mice, exhibiting significantly better spirits, shiny color, and appetite than the CTX-administered mice.

### 3.5. TFSD and FMN Alleviated CTX-Induced Injury in Immune Organs

**Figure 4:**
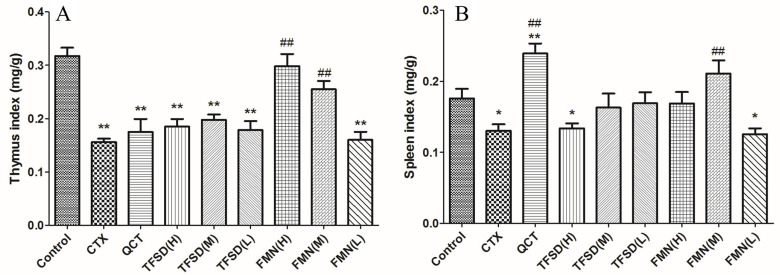
Effects of TFSD and FMN on the organ index of mice treated with CTX. (**A**) Thymus index. (**B**) Spleen index. Data represent mean ± SEM (n = 10). * indicates a significant difference from the control group (*p* < 0.05), while ** denotes a highly significant difference (*p* < 0.01). ## indicates a highly significant difference (*p* < 0.01).

CTX treatment induced severe splenic and thymic atrophy, significantly reducing organ indices (*p* < 0.05). Compared with the CTX treatment, TFSD and FMN pretreatment inhibited the atrophy of the spleen and thymus, exhibiting significantly higher spleen and thymus indexes. Mice were pretreated with FMN in doses of 50 and 25 mg/kg.bw exhibited significantly higher thymus index (*p* < 0.01), indicating a better preventative effect than the positive control of QCT. Regarding the spleen index, 25 mg/kg.bw of FMN exhibited the best effect in preventing the atrophy of the spleen, revealing a similar effect as that of QCT (Figure 4).

**Figure 5:**
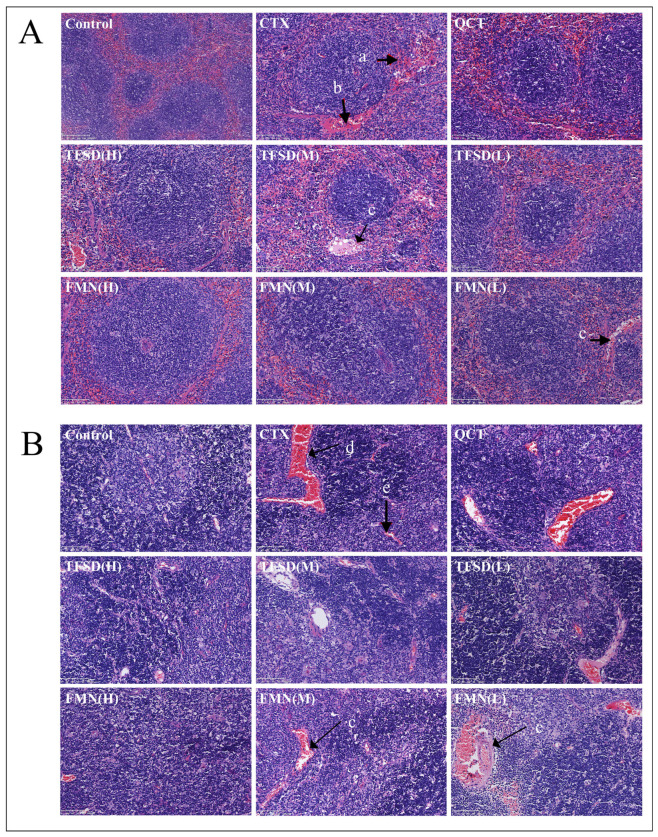
H&E staining examination of the effect of TFSD and FMN on CTX-induced pathological injury in the spleen and thymus. (H&E stain, 100×). (**A**) Spleen. (**B**) Thymus. a: splenic capillary dilatation and congestion. b: diffuse hemorrhage from a splenic mass. c: slight congestion. d: erythrocytic infiltration of the thymus and structural destruction of the thymic lobules, and e: decreased number of LYM in the thymic lobules.

H&E-stained sections revealed CTX-induced splenic and thymic damage, including disrupted splenic corpuscle architecture and peri-corpuscular ‘Red Halo’ formation (Figure 5). Additionally, dilatation and hyperemia of splenic capillaries, and diffuse hemorrhage in splenic corpuscles were observed. Infiltration of RBC and destruction of thymus lobule structure were observed in the thymus of CTX-treated mice, exhibiting a decreased number of LYM in the thymus lobules. However, pretreatment with TFSD or FMN for seven consecutive days significantly reduced CTX-induced pathological damage in both the spleen and thymus.

### 3.6. TFSD and FMN Modulated Biomarker Levels in the Blood Routine

**Figure 6:**
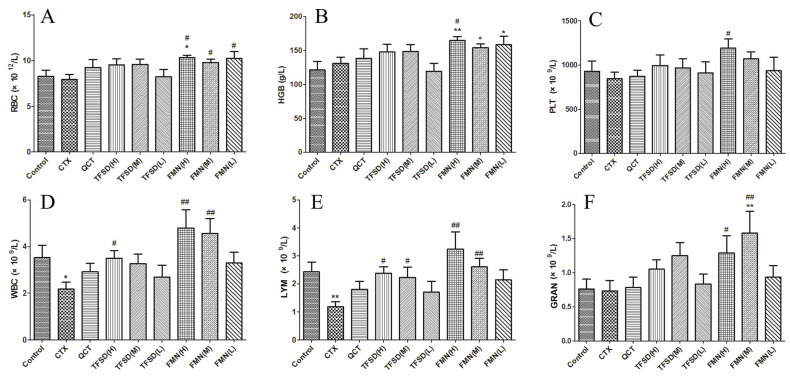
Blood routine analysis of the effect of TFSD and FMN on CTX-induced fluctuation of blood components. (**A**) Red blood cells (RBC). (**B**) Hemoglobin (HGB). (**C**) Platelets (PLT). (**D**) White blood cells (WBC). (**E**) Lymphocytes (LYM). (**F**) Neutrophilic granulocyte count (GRAN). Data represent mean ± SEM (n = 10). * indicates a significant difference from the control group (*p* < 0.05), while ** denotes a highly significant difference (*p* < 0.01). # represents a significant difference compared to the model CTX group (*p* < 0.05), and ## indicates a highly significant difference (*p* < 0.01).

The hematological analysis indicated (Figure 6) that CTX treatment altered the number of RBC, HGB, PLT, and GRAN compared to the control group; however, these differences were not statistically significant (*p* > 0.05). However, FMN pretreatment significantly increased the levels of these indexes (*p* < 0.05), indicating that FMN can be an effective component in promoting hematopoiesis. The WBC and Lymph counts significantly decreased after CTX administration (*p* < 0.05). The reduced quantity of WBC and LYM cells, which are crucial for pathogen defense, suggests that CTX can compromise immunity by reducing the number of immune cells in the blood circulation system. TFSD and FMN pretreatments inhibited the CTX-induced reduction in WBC and LYM, and higher doses of FMN exhibited the best preventative effect compared to TFSD and low doses of FMN.

### 3.7. TFSD and FMN Promote Secretion of Immunoglobulins and Key Cytokines

**Figure 7:**
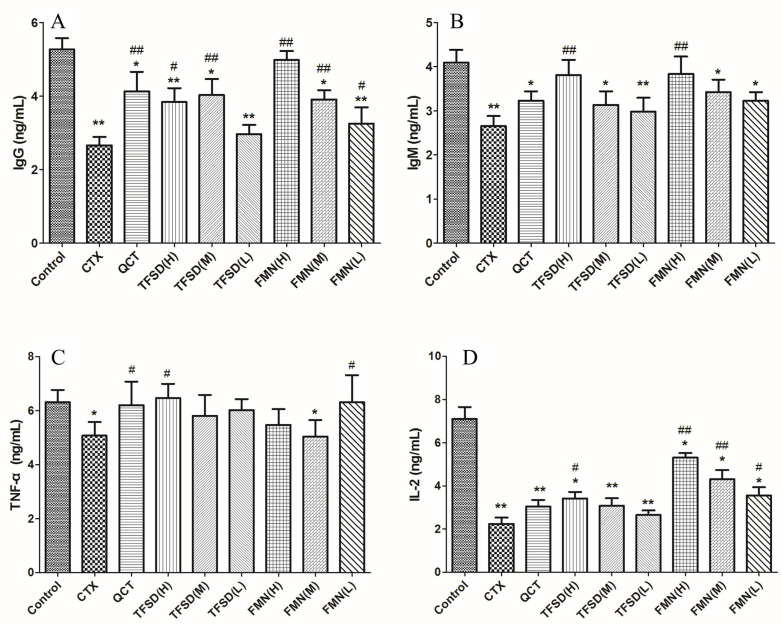
Measurement of the effects of TFSD and FMN on the production of immunoglobulins and key cytokines in mice dosed with CTX through ELISA. (**A**) IgG. (**B**) IgM. (**C**) TNF-ɑ. (**D**) IL-2. Data represent mean ± SEM (n = 10). * indicates a significant difference from the control group (*p* < 0.05), while ** denotes a highly significant difference (*p* < 0.01). # represents a significant difference compared to the model CTX group (*p* < 0.05), and ## indicates a highly significant difference (*p* < 0.01).

CTX treatment significantly decreased the IgM, IgG, TNF-α, and IL-2 serum levels in mice (*p* < 0.05 or *p* < 0.01). However, pre-treatment with TFSD or FMN significantly increased the levels of IgG and IgM (*p* < 0.05, Figure 7A,B). In CTX-treated mice, the most effective doses for promoting immunoglobulin production were 100 mg/kg.bw TFSD and 50 mg/kg.bw FMN. TFSD (100 mg/kg.bw) or FMN (12.5 mg/kg.bw) prevented the abnormal changes in TNF-α (*p* < 0.05, Figure 7C). In addition, high doses of TFSD (100 mg/kg.bw) and FMN (50, 25, 12.5 mg/kg.bw) significantly increased the levels of IL-2 in CTX-induced immunosuppressed mice (*p* < 0.05, Figure 7D). IL-2 was best modulated by, the high dose of FMN compared to TFSD and lower doses of FMN.

### 3.8. TFSD and FMN Regulated the mRNA Expression of Key Cytokines in the Spleen

**Figure 8:**
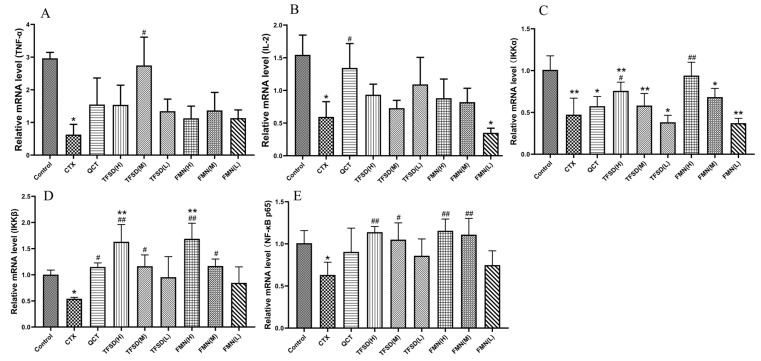
The effects of TFSD and FMN on the expression of key cytokines in the spleen. (**A**) TNF-ɑ. (**B**) IL-2. (**C**) IKKα. (**D**) IKKβ. (**E**) NF-κB p65. Data represent mean ± SEM (n = 10). * indicates a significant difference from the control group (*p* < 0.05), while ** denotes a highly significant difference (*p* < 0.01). # represents a significant difference compared to the model CTX group (*p* < 0.05), and ## indicates a highly significant difference (*p* < 0.01).

The relative mRNA expression of key cytokines in splenic tissue was measured. The CTX group exhibited a significant reduction in the mRNA expression levels of IL-2, TNF-α, IKKα, IKKβ, and NF-κB p65 (*p* < 0.05 or *p* < 0.01) compared to the control group. Conversely, in comparison to the CTX group, pretreatment with TFSD and FMN significantly upregulated the mRNA expression of TNF-α, IL-2, IKKα, IKKβ, and NF-κB p65 (*p* < 0.05 or *p* < 0.01, Figure 8).

## 4. Discussion

Studies have demonstrated that *S. suberectus Dunn* enhances immune function and exhibits a range of pharmacological activities, including antioxidant, antiviral, antitumor, and regulation of lipid metabolism [18]. Consequently, *S. suberectus Dunn* holds significant potential for treating immunosuppressive diseases. This herb is rich in flavonoids, which are essential for enhancing the immune system and anti-inflammatory responses [36]. Network pharmacology analysis of *S. suberectus Dunn* identified its top ten major active ingredients, including flavonoids FMN, vestitol, and psi-baptigenin. These results indicate that TFSD, the primary component of *S. suberectus Dunn*, is likely responsible for its effects against immunosuppression. Metabolomic analysis revealed a total of 2077 metabolites, with flavonoids accounting for 501 of these metabolites. FMN exhibited the highest relative content, suggesting that FMN can be the primary active ingredient responsible for the pharmacological effects of TFSD. Furthermore, component-target network and PPI network analyses indicated that *S. suberectus Dunn* can counteract immunosuppression by regulating key targets, including NF-κB p65 and IKKβ. NF-κB p65 and IKK are essential for activating the classical NF-κB signaling pathway [37]. Combined with GO enrichment and KEGG pathway analyses, the NF-κB signaling pathway was the most significantly implicated, with the smallest *p*-value and largest count value. This pathway is essential for the regulation of inflammation and proliferation, which further suggests that *S. suberectus Dunn* can mitigate immunosuppression by modulating the NF-κB signaling pathway.

CTX is an alkylating agent and cytotoxic drug extensively used in tumor treatment, including lymphoma in dogs and cats. It has a strong immunosuppressive effect in animals [38,39]. CTX is frequently employed in scientific research utilized to establish immunosuppression models for evaluating the immunomodulatory activity of therapeutic candidates [40]. To further investigate the anti-immunosuppressive mechanism of *S. suberectus Dunn*, we established an immunosuppressive mouse model through intraperitoneal administration of 200 mg/kg CTX. The results were consistent with previous reports on CTX-induced immunosuppression. In the CTX-treated group, thymic and splenic indices were significantly reduced, thymic lobules were destroyed, and splenic vesicles exhibited diffuse hemorrhages [41]. Additionally, WBC and LYM [42], the levels of IgM and IgG [43], and serum TNF-α and IL-2 levels were significantly reduced [44]. Accordingly, a successful immunosuppressed model was established using CTX. TFSD and FMN likely modulate CTX-induced immunosuppression by regulating the NF-κB signaling pathway. This was demonstrated by their ability to attenuate CTX-induced pathological injury in the spleen and thymus, increase immune cell counts, enhance immunoglobulin production, and upregulate the secretion of TNF-α and IL-2.

As primary immune organs, the thymus and spleen mediate T cell activation and antibody production [2]. Additionally, the thymus promotes mast cell development, while the spleen facilitates GRAN phagocytosis during immune responses [45]. In this study, immunosuppression in CTX-treated mice was confirmed by significant atrophy of spleen and thymus tissues and decreased organ indices. Histological analysis of H&E staining exhibited dilated and hyperemic splenic capillaries, diffuse hemorrhage in the splenic corpuscles, and destruction of thymus lobules, indicating immune organ injury in CTX-treated mice. Furthermore, the number of lymphoid nodules and splenic vesicles decreased and became smaller, suggesting that CTX affected the production of immune cells in mice. Pretreatment with TFSD and FMN alleviated CTX-induced damage by inhibiting organ atrophy and preventing structural damage to the spleen and thymus. These results are consistent with the findings from studies suggesting that total flavonoids from *Pteris multifida Poir* and persimmon leaf extracts can improve thymus and spleen indices in CTX-treated mice, stimulate immune organ development, and enhance overall immune function [46,47].

B cells mature in the bone marrow and produce key antibodies (IgM and IgG) essential for humoral immunity [48]. In clinical chemotherapy, CTX-induced myelosuppression is a major concern for cancer patients. Consistent with prior studies, we observed a significant reduction in immune cell counts, including WBC, LYM, and GRAN, in CTX-treated mice [42]. This reduction in immune cell populations resulted in a decrease in the production of immunoglobulins, impairing the body’s ability to neutralize pathogens. This immunosuppression was likely due to the atrophy and damage of immune organs mentioned earlier. In this study, pretreatment with TFSD and FMN significantly increased immune cell counts and immunoglobulin levels. Additionally, QCT increased the spleen index and serum IgM levels in CTX-treated mice, indicating that TFSD, FMN, and QCT enhance immune responses and protect against pathogen invasion. Studies have suggested that icaritin, a flavonoid, can improve spleen and thymus indices and promote hematopoietic stem cell proliferation, thereby alleviating CTX-induced myelosuppression [49]. The potential of TFSD and FMN to protect against CTX-induced myelosuppression and immunosuppression was further supported by the observation of similar effects in our study.

Cytokines, secreted by both immune (T/B/NK cells) and non-immune cells (endothelial/epithelial cells), are crucial immunoregulators. They mediate immune functions by activating immune cells and promoting the release of immunoglobulins [50,51]. IL-2 enhances the killing activity of T cells, stimulates NK cells, induces TNF-α and IFN-γ secretion, and promotes B cell proliferation and immunoglobulin production [52]. TNF-α activates monocytes and macrophages, increasing their killing activity and antigen presentation [53]. In CTX-treated mice, we observed reduced secretion and mRNA expression of TNF-α and IL-2, which is consistent with previous studies [54,55]. Pretreatment with TFSD and FMN inhibited these changes in cytokine secretion and mRNA expression, indicating that they protect against CTX-induced immune injury by regulating both cellular and humoral immune responses. FMN exhibited a more potent preventive effect compared to TFSD, with a high dose (50 mg/kg.bw) yielding the best results in most parameters. These data suggest that FMN is a key active component in TFSD and can be a crucial raw material in *S. suberectus Dunn*.

The NF-κB signaling pathway regulates cytokine synthesis and release, playing a pivotal role in immune modulation with broad physiological and pathological implications [56,57,58]. NF-κB p65 remains sequestered in the cytoplasm through its interaction with IκBα under physiological conditions. Upon activation, IKK (composed of IKKα, IKKβ, and IKKγ) phosphorylates and degrades IκBα, releasing NF-κB p65 to initiate inflammatory responses [59]. TFSD and FMN alleviate immunosuppression-induced inflammatory injury by activating the NF-κB pathway, specifically through upregulation of NF-κB p65, IKKα, and IKKβ mRNA expression. These results are consistent with previous reports that CTX-induced immunosuppression can be alleviated by Ginseng-DF upregulating MAPK/NF-κB expression [60]. Furthermore, FMN exhibited a stronger protective effect than TFSD and QCT, with the high dose (50 mg/kg) demonstrating the most significant improvements. These findings further suggest that FMN is a primary active component of TFSD and *S. suberectus Dunn* and can help treat CTX-induced immunosuppression by regulating the NF-κB signaling pathway, cytokine secretion, and immune cell function.

However, this study has certain limitations. Antimicrobial properties of *S. suberectus Dunn* flavonoids (FMN) could alter microbial composition, as observed with other flavonoid-rich extracts (*Scutellaria baicalensis* flavonoids reducing pathogenic *Helicobacteraceae* while promoting beneficial Lactobacillus) [61]. Microbiota-immune crosstalk can synergize with NF-κB modulation. In addition, flavonoid induction increases in short-chain fatty acid-producing bacteria (*Muribaculaceae*), which can promote regulatory T-cell differentiation, further alleviating immunosuppression [62]. TFSD and FMN can reduce CTX-induced immunosuppression in mice through NF-κB pathway modulation; however, further validation using specific inhibitors is required. Additionally, complementary in vitro studies are required to confirm their regulatory effects on NF-κB signaling.

## 5. Conclusions

Metabolomics, network pharmacology, and molecular docking analyses identified the NF-κB signaling pathway as the primary mechanism through which *S. suberectus Dunn* regulates immunosuppression. Flavonoids, the primary active components of *S. suberectus Dunn*, with FMN as the key active ingredient in TFSD, were observed to mediate these effects. TFSD and FMN improved the immunosuppressed state in mice, as indicated by increased organ indices, reduced pathological damage in the spleen and thymus, increased immune cell populations (leukocytes, LYM, and GRAN), and elevated production of immunoglobulins (IgM and IgG). Additionally, both compounds upregulated the secretion and expression of critical cytokines. TFSD and FMN can protect mice from CTX-induced immunosuppression by regulating the NF-κB signaling pathway, making them promising drug candidates for preventing and treating immunosuppression-related diseases.

## Figures and Tables

**Table 1:** Grouping and treatment of mice.

Group	D1 to D7	D7
Control	PBS 0.2 mL/mouse	PBS 0.2 mL/mouse
CTX	PBS 0.2 mL/mouse	CTX 200 mg/kg.bw
QCT	QCT 100 mg/kg.bw	CTX 200 mg/kg.bw
TFSD(H)	TFSD 100 mg/kg.bw	CTX 200 mg/kg.bw
TFSD(M)	TFSD 50 mg/kg.bw	CTX 200 mg/kg.bw
TFSD(L)	TFSD 25 mg/kg.bw	CTX 200 mg/kg.bw
FMN(H)	FMN 50 mg/kg.bw	CTX 200 mg/kg.bw
FMN(M)	FMN 25 mg/kg.bw	CTX 200 mg/kg.bw
FMN(L)	FMN 12.5 mg/kg.bw	CTX 200 mg/kg.bw

Note: CTX: cyclophosphamide. QCT: quercetin. TFSD: total flavonoid of *Spatholobus suberctus Dunn*. FMN: formononetin. PBS: phosphate-buffered saline. D1: day 1. D7: day 7. H, M, and L represent high, medium, and low doses, respectively. QCT, TFSD, FMN, and CTX were dissolved in PBS (10 mM, pH 7.4). Mice received daily intragastric administration (0.2 mL/day × 7 days) of PBS, QCT, TFSD, or FMN, with concurrent intraperitoneal injections of normal saline or CTX (0.2 mL) on day 7. Several procedural adaptations were incorporated to mitigate potential confounding effects of handling-induced stress during compound administration: a. Operators are trained until >95% accuracy is achieved in the dye distribution test. b. Fast for 4 h prior to dosing (no water) to reduce the effect of gastric contents on drug absorption. A sterile blunt stainless steel gavage needle (diameter 1.2 mm, length 3 cm) was used, covered with a silicone tube to prevent esophageal damage. c. Adjust the dosing volume (usually 10 mL/kg) according to the body weight of the mouse (e.g., 20 g) to ensure that the actual dosage error of each animal is <5%.

**Table 2:** Primer sequences for RT-qPCR analysis of target genes.

Gene	Primers Sequences (5′-3′)	Products Size (bp)	Accession Number
β-actin	F: 5′-ATCACTATTGGCAACGAGCG-3′	191	NM_007393.5
	R: 5′-TCAGCAATGCCTGGGTACAT-3′		
TNF-α	F: 5′-AGCACAGAAAGCATGATCCG-3′	212	NM_013693.3
	R: 5′-CTGATGAGAGGGAGGCCATT-3′		
IL-2	F: 5′-GATGGATAGCCTTCTGTC-3′	82	NM_008366.3
	R: 5′-GAGAGCCTTATGTGTTGT-3′		
IKKα	F: 5′-AGTTCTGCCCGCTCTCTTGTAG-3′	100	XM_030250732.2
	R: 5′-GAGGATGTTCACGGTCTGCTAATG-3′		
IKKβ	F: 5′-GCAGAAGAGCGAAGTGGACATC-3′	112	NM_001424831.1
	R: 5′-CAGCCGTTCAGCCAAGACAC-3′		
NF-κB p65	F: 5′-GACCTGGAGCAAGCCATTAG-3′	125	NM_001402548.1
	R: 5′-CGCACTGTCACCTGGAAGC-3′		

**Table 3:** Top 10 metabolites in *S. suberectus Dunn* by relative content.

Sample	Class I	*S. suberectus Dunn* 1	*S. suberectus Dunn* 2	*S. suberectus Dunn* 3
Formononetin	Flavonoids	5.32 × 10^7^	6.16 × 10^7^	5.80 × 10^7^
Isoliquiritigenin	Flavonoids	4.65 × 10^7^	4.79 × 10^7^	4.64 × 10^7^
Quercetin	Flavonoids	3.60 × 10^7^	3.16 × 10^7^	3.49 × 10^7^
Medicagol	Flavonoids	3.45 × 10^7^	3.46 × 10^7^	3.42 × 10^7^
Peganone I	Quinones	3.18 × 10^7^	2.19 × 10^7^	2.68 × 10^7^
Stearic Acid	Lipids	3.14 × 10^7^	5.27 × 10^7^	4.54 × 10^7^
3′,7-dihydroxy-4′-methoxyflavone	Flavonoids	2.85 × 10^7^	2.53 × 10^7^	2.57 × 10^7^
L-Pipecolic Acid	Alkaloids	2.77 × 10^7^	3.01 × 10^7^	1.93 × 10^7^
Protogenkwanone	Flavonoids	2.73 × 10^7^	2.66 × 10^7^	2.85 × 10^7^
5,7,2′-Trihydroxy-8-methoxyflavone	Flavonoids	2.68 × 10^7^	2.62 × 10^7^	2.78 × 10^7^

**Table 4:** Components of *S. suberectus Dunn* (top 10 of OB value).

Mol ID	Component	OB (%)	DL
MOL000483	(Z)-3-(4-hydroxy-3-methoxy-phenyl)-N-[2-(4-hydroxyphenyl)ethyl]acrylamide	118.35	0.26
MOL000471	aloe-emodin	83.38	0.24
MOL000500	Vestitol	74.66	0.21
MOL000468	8-o-Methylreyusi	70.32	0.27
MOL000507	Psi-Baptigenin	70.12	0.31
MOL000392	Formononetin	69.67	0.21
MOL000502	Cajinin	68.8	0.27
MOL000501	Consume close grain	68.12	0.27
MOL000506	Lupinidine	61.89	0.21
MOL000503	Medicagol	57.49	0.6

## Data Availability

All data are available from the corresponding author by request.

## Abbreviations

The following abbreviations are used in this manuscript:

TFSD	Total flavonoid of *Spatholobus suberctus Dunn*
CTX	Cyclophosphamide
NF-κB	Nuclear factor kappa-B
IKK	I Kappa B Kinase
QCT	Quercetin
TNF-α	Tumor necrosis factor-ɑ
IgG	immunoglobulin G
IgM	immunoglobulin M
IL-2	Interleukin-2
GO	Gene ontology
KEGG	Kyoto encyclopedia of genes and genomes
BP	Biological processes
CC	Cellular components
MF	Molecular functions
TIC	Total ion current
QC	Quality control
OB	Oral bioavailability
DL	Drug-likeness
PPI	Protein–protein interaction
